# Factors influencing general practitioners’ perception of and attitude towards dementia diagnostics and care—results of a survey among primary care physicians in Germany

**DOI:** 10.1007/s10354-020-00803-9

**Published:** 2021-01-14

**Authors:** Julian Wangler, Michael Jansky

**Affiliations:** Centre for General Medicine and Geriatrics, Universitätsmedizin Mainz, Am Pulverturm 13, 55131 Mainz, Germany

**Keywords:** Dementia care, General practitioner, Dementia diagnosis, Early detection, Attitudes and perceptions, Demenzversorgung, Hausarzt, Demenzdiagnostik, Früherkennung, Wahrnehmungs- und Handlungsmuster

## Abstract

Studies have shown that primary care is not always effective when it comes to caring for people with dementia. In addition, general practitioners do not always use diagnostic instruments consistently. The aim of the study was to identify relevant factors that influence general practitioners’ attitudes and willingness with respect to consistent diagnosis and care. For this purpose, resources, viewpoints, and behavioral patterns of general practitioners with regard to dementia diagnostics as well as common challenges in everyday practice were recorded. In the course of a survey, a total of 2266 general practitioners in Hesse and Baden-Württemberg were interviewed between January and March 2020. In addition to the descriptive analysis, a *t*-test was used to determine significant differences between two groups. A univariate linear regression analysis was carried out to identify possible influencing factors. 81% of the respondents do provide dementia diagnostics; 51% are involved in the treatment. Most of them see the diagnostic work-up (77%), communication and compliance problems (73%), as well as the therapeutic support (71%) as common challenges. In addition, there are interface problems regarding the interdisciplinary cooperation. Some of the respondents express doubts about the value of early detection (41%). The general practitioners’ attitude with respect to dementia diagnostics and care is determined by influencing factors that relate to geriatric competencies, expectations of self-efficacy, the integration of practice staff, as well as the knowledge of and cooperation with counseling and care services. It seems advisable to strengthen the geriatric competence of general practitioners. Moreover, it appears essential to educate general practitioners more about support structures in the field of dementia care and to integrate them accordingly. In addition, practice staff should be more systematically involved in the identification and care of dementia patients.

**Supplementary Information:**

The online version of this article (10.1007/s10354-020-00803-9) contains supplementary material, which is available to authorized users.

## Abstract (de)

Studien haben gezeigt, dass die hausärztliche Demenzversorgung Mängel aufweist und Allgemeinmediziner beim Einsatz diagnostischer Verfahren nicht immer konsequent agieren. Ziel der vorliegenden Studie war es, relevante Faktoren zu identifizieren, die die Einstellung und Bereitschaft zur Demenzdiagnostik und -versorgung beeinflussen. Dazu wurden Ressourcen, Haltungen und Verhaltensweisen von Hausärzten in Bezug auf die Demenzdiagnostik sowie im Praxisalltag verbreitete Hausforderungen erfasst. Im Zuge einer schriftlichen Befragung wurden zwischen Januar und März 2020 insgesamt 2266 Hausärzte in Hessen und Baden-Württemberg befragt. Neben der deskriptiven Analyse kam zur Feststellung von signifikanten Unterschieden zwischen 2 Gruppen ein t‑Test bei unabhängigen Stichproben zum Einsatz. Zur Identifizierung möglicher Einflussfaktoren wurde eine univariate lineare Regressionsanalyse durchgeführt. Eine Demenzdiagnostik bieten 81 % der Befragten an; 51 % sind bei der Therapie eingebunden. Als verbreitete Herausforderungen werden neben diagnostischer Abklärung (77 %) kommunikative und Compliance-Probleme (73 %) sowie therapeutische Begleitung (71 %) genannt. Zudem kommt es in der interdisziplinären Zusammenarbeit zu Schnittstellenproblemen. Ein Teil der Befragten bringt Zweifel am Wert einer Früherkennung zum Ausdruck (41 %). Die hausärztliche Einstellung in Bezug auf die Demenzversorgung wird durch Einflussfaktoren mitbestimmt, die sich auf geriatrische Kompetenzen, Selbstwirksamkeitserwartungen, die Integration des Praxispersonals sowie die Kenntnis von und Kooperation mit Hilfs- und Unterstützungsangeboten beziehen. Es erscheint ratsam, die geriatrische Kompetenz von Hausärzten zu stärken. Außerdem scheint es wichtig, Hausärzte mehr über Unterstützungsstrukturen im Bereich der Demenzversorgung zu informieren und sie darin einzubeziehen. Darüber hinaus sollte das Praxispersonal systematischer an der Erkennung und Versorgung von Demenzpatienten beteiligt werden.

In light of the presented results, it would seem necessary to strengthen the geriatric competencies of general practitioners. Furthermore, general practitioners must be better informed about support structures in dementia care, and be integrated into these concepts. Moreover, practice staff should be more systematically involved in the identification and treatment of dementia patients.

## Introduction

General practitioners have often known patients for years who come to their consultations on a regular basis, so the general medical practice setting provides favorable conditions for identifying changes in cognitive condition in a timely manner [1–3]. This gives general practitioners a key role in identifying and treating patients with dementia [4, 5]. Whether a general practitioner refers a patient to a specialist or memory clinic and/or makes a diagnosis on the spot at the time the patient is suspected of cognitive impairment calling for diagnosis also plays an important part [6].

Even so, studies have shown deficits in primary care in treating dementia patients [7–10]. For example, there is evidence of lack of knowledge of guidelines and treatment options [11–13], sufficient diagnostics or exclusion diagnostics [14]. Studies have also shown general practitioners to be relatively unwilling to use dementia tests early and consistently [15]. Apart from that, one study in a model project for outpatient dementia care has shown that most general practitioners prefer to leave diagnosis (according to guidelines) to specialists [6]. Two interview studies found that a significant number of responding general practitioners tended to refer dementia patients to specialists as fast as possible and scarcely participated in the healthcare process [16, 17].

Despite these findings, comparatively few studies have researched the causes of this reluctance on the part of many general practitioners to treat dementia patients [18]. This especially applies to the German-speaking world. Several international systematic reviews have claimed a “lack of training and confidence” on the part of general practitioners in treating dementia patients; this was reinforced by system-related barriers, especially “a lack of time during consultations and lack of support services” [19]. There are also signs of communicative uncertainty in diagnosing dementia [20]. Qualitative studies suggest that the reluctance of general practitioners to use dementia tests is part of a complex of corresponding factors [21]. Reasons included time pressure and shortage of resources as well as low expectations on efficacy due to the perceived lack of treatment options [4], fear of stigmatizing patients, and cultural factors [16, 21–24].

There is still a lack of studies that have attempted to identify relevant factors that may play a substantial role in the willingness of general practitioners to apply dementia diagnostics and treatment. This survey was aimed at elucidating these conditions and predictors for the quality and effectiveness of dementia treatment given by general practitioners. The primary research interest can therefore be summarized in the following issues:

- What perceptions and attitudes prevail amongst general practitioners with regard to dementia diagnostics and treatment?
- What determines the willingness of general practitioners to provide dementia diagnostics consistently and participate in managing the treatment of dementia patients?

## Materials and methods

### Study design and setting

This study was based on a pre-study from the year 2018 in which the questionnaire used was pretested for concept amongst 425 general practitioners in the Rhineland-Palatinate federal state [25]. Especially the German-speaking world still lacks studies addressing the attitudes of general practitioners to dementia diagnostics and their causes in a thorough way. The survey was therefore brought up to date and repeated on a larger scale. One of the aims was to test whether the results from that time could be confirmed. Another aim was to gather a dataset large enough to enable more in-depth analysis of possible influential factors.

### Questionnaire and sociodemographic variables

The aim of the survey was to develop a broad picture of the situation considering indicators for a specific pattern of attitudes and actions in dementia treatment in primary care. Indicators towards identifying the roles that general practitioners saw themselves in while identifying and treating dementia patients were studied alongside the relevant challenges and hurdles in treating dementia as well as in interdisciplinary cooperation.

As previously mentioned, the questionnaire was based on the previous survey study [25] and is also supported by results from several exploratory studies [16, 17] based on semi-standardized interviews with general practitioners. These studies have identified different general practitioner types with clear distinctions between treatment of dementia patients, use of dementia diagnostics, and their own perceived role in the topic.

The questionnaire (see Supplementary Information; time to complete: 10–12 min) addressed the following items: attitudes towards dementia as a disease; specialist diagnostic expertise; use and analysis of existing testing methods; communication with patients and family members; general practice management; networking with other support services; challenges experienced; subjective assumptions of effectiveness; and interest in specific further training.

Sociodemographic data included age, gender, type of medical practice, and the number of doctors and patients per quarter. Respondents were also asked to give a rough classification of the population of the town in which their medical practice was located as well as further training in geriatrics^1^.

A pretest was carried out before the actual survey. The questionnaire was presented to a total of 15 general practitioners in order to check the comprehensibility and completeness of the categories. As it turned out, there were no major problems in processing the questionnaires.

### Recruitment and participants

The anonymous written postal survey was conducted between 15 January and 31 March 2020. All 3839 active general practitioners in Hesse and 6664 active general practitioners in Baden-Württemberg were invited to take part in the survey. Participants were not given any remuneration.

### Sampling

A total of 2266 completed questionnaires out of 2315 processed were included in analysis at a total response rate of 22%^2^. The sample was structured as follows:

- Gender: 55% male, 45% female
- Office setting: 37% in medium-sized and large towns or cities, 63% in small towns or rural areas
- Type of office: 59% individual doctor’s offices, 39% joint offices, 2% other
- Patients per quarter: 24% < 1000, 33% 1000–1500, 43% > 1500
- Mean age: 55 years
- Further training in geriatrics: 33%

### Ethics

During this study, no sensitive patient data were gathered or clinical tests performed. This is a strictly anonymized survey of a total of 2266 general practitioners. However, the authors of the study contacted the Ethics Commission of the State of Rhineland-Palatinate before beginning the study to ensure that it conformed with the medical professional code of conduct.

### Data analysis

After cleansing, we analyzed the data using SPSS 23.0 for Windows. We used a *t*-test for independent samples to analyze for any significant differences between the two groups, assuming significance at values of *p* ≤ 0.001. Unlike the preliminary study, the analysis focused on the primary research interest of identifying possible influential factors using univariate linear regression analysis at a significance level of *p* ≤ 0.05. This eliminated aspects of the questionnaire without any direct relationship in the following presentation of results.

## Results

### Diagnostics and interdisciplinary cooperation

In response to the question about patients who had shown (incipient) dementia or suspicion of dementia, 89% of the respondents stated that the first indications that suggested possible dementia came from family members, 71% quoted their own dealings with the patient, whereas half of the respondents (51%) mentioned complaints from patients themselves and 38% took their evidence for potential dementia from other staff members in the practice. Doctors with further training in geriatrics took information from their staff on board at 51%, compared to 30% amongst doctors without corresponding training (α = 0.001; *p* = 0.0).

The general practice provided dementia diagnostics according to 81% of general practitioners. Just under one in every five general practitioners stated that they had abandoned dementia diagnostics (11%) or had never offered it in the first place (8%). The methods most commonly used were the clock test at 75% followed by the Mini Mental Status Test at 71% and DemTect at 60%. A greater share of respondents without any dementia diagnostics in their own general practice felt substantially less capable of diagnosing dementia on time compared to the other respondents (63% compared to 16%; α = 0.001; *p* = 0.0).

While 70% of respondents stated that they used tests only on suspicion of dementia or in follow-up, 38% made use of the tests during general geriatric assessment. Only 29% of the respondents used dementia tests specifically for screening purposes except for the GBA (Federal Joint Committee). One striking result was that physicians with further training in geriatrics screened their patients far more often (42%) than did those without this background (21%; α = 0.001; *p* = 0.0).

Just over one in three of the general practitioners (34%) had practice staff who had undergone corresponding further training. There were no correspondingly qualified practice staff members in 66% of cases. The proportion of trained practice staff in practices run by general practitioners with training in geriatrics was more than double that of general practitioners without adequate further training (52% vs. 21%; α = 0.001; *p* = 0.0). Physicians with staff trained in dementia stated more often that indications as to a potential dementia condition come from their staff members (44% vs. 35%).

Just over half the respondents (52%) stated that they were working with specific organizations or services involved in supporting and caring for dementia patients and their family members (42% said they were not, 6% gave no answer). An open question showed that these were mostly outpatient and inpatient care services. Exactly half the respondents (50%) claimed a good level of familiarity with regional support structures such as dementia networks and care centers (47% claimed not so much familiarity or none at all).

Almost nine out of ten general practitioners (89%) responded that they always referred patients suspected of dementia or similar diagnosis to a specialist. Of these, 84% referred patients to neurologists or psychiatrists while 24% referred them to a memory clinic. Just under 11% of all respondents stated that they would not usually refer patients suspected of dementia.

Just over half the general practitioners (51%) responded that they were involved in treating their dementia patients and took on corresponding treatment activities in consultation with specialists. Exactly a quarter of all respondents (25%) left dementia treatment to specialists. Just under a quarter (24%) only involved themselves in treatment in individual or exceptional cases.

### Challenges in procedures at the practice

Under half the respondents (47%) found it difficult or very difficult to identify incipient dementia in their patients, whereas more than half the respondents (53%) did not see any particular challenge in identifying dementia. Respondents who had abandoned or had never had dementia diagnostics at their general practice saw diagnosing dementia as a challenge more often than the other respondents (70% vs. 42%; α = 0.001; *p* = 0.0).

The respondents were presented with a set of items with potential challenges later on in the proceedings. Apart from cost-covering consultation and treatment of dementia patients (80%), especially differential diagnostic evaluation (77%) is considered to be particularly difficult, followed by communications and compliance issues while briefing patients on their diagnosis (73%). At 71%, the great majority of respondents also saw successful treatment of patients as challenging or very challenging.

Several quotations were taken from a qualitative preliminary study [22] in the form of a short set for agreement or disagreement (see Table 1) in order to bring more depth into the challenges involved. Apart from the issues involved in ensuring a financially viable diagnostic method for dementia, a substantial number of respondents reported severe resistance from patients in response to the diagnosis, which limited further treatment. A similarly high number of respondents perceived a lack of treatment options in dementia and therefore questioned the value of early identification. One of the results of these hurdles in general practice was that a third of the respondents shared the opinion that treating dementia patients should be left to specialists.

**Table 1:** This table shows a variety of statements from general practitioners that were collected during the interview study. How strongly do you agree with each statement? (*N* = 2266)

Statement	Completely agree/somewhat agree (%)
1) “*There is little financial incentive for general practitioners to treat dementia; the fees need to be significantly higher.”*	72.3
2) *“To many patients, diagnosing dementia is an affront to their ability to make decisions independently. I’m extremely reluctant to do this, and this restricts for the patient care.”*	43.6
3) *“There are no real benefits to diagnosing dementia early considering the insufficient treatment options.”*	41.4
4) *“Dementia patients should always be treated by a specialist such as a neurologist or psychiatrist, not by a general practitioner.”*	31.2

More than half the general practitioners who had withdrawn from diagnosing dementia were of the opinion that treating dementia patients was the responsibility of a specialist (56%) compared to only 24% amongst general practitioners providing demented diagnostics in their practice (α = 0.001; *p* = 0.0). Another finding was that far fewer general practitioners with further training in geriatrics doubted the implied benefit of early diagnosis in the corresponding item compared to their colleagues without adequate further training (agreement to statement 3: 33% vs. 45%). Conversely, fewer respondents who no longer provided dementia diagnostics saw benefits in early diagnosis compared to other respondents (agreement to statement 3: 48% vs. 39%).

Respondents named common challenges in communication and cooperation between general practitioners and specialists regarding outpatient examination and treatment in dementia (see Table 2). Most respondents mentioned problems scheduling appointments with specialists as well as interdisciplinary communication in routine practice. Two-thirds of the respondents had the impression that specialists did not brief patients enough about their situation, and also that specialist findings reached the general practitioners treating the patients too late.

**Table 2:** In your experience, how often do the following difficulties arise? (*N* = 2266)

Statement	Often (%)	Sometimes (%)
Neurologists and psychiatric specialists are booked up long into the future	86.1	12.0
We do not have the time to discuss mostly complex patient problems with colleagues in routine practice	75.0	19.8
Neurologists and psychiatrists are difficult for patients to reach	72.0	22.5
There are too few neurological and psychiatric practices nearby	61.4	19.3
Neurologists and psychiatrists do not brief patients enough, who then go back to general practitioners out of uncertainty	24.2	44.2
It takes too long for neurologists and psychiatrists to pass on their findings	29.0	39.1
Neurologists and psychiatrists do not inform general practitioners enough about the tests they have conducted or the results and/or diagnoses they have made	16.2	41.6

### Factors influencing general practitioners in diagnostics and treatment

Results from univariate linear regression analysis reveal a series of stronger and weaker influential factors for central variables (see Table 3). Subjective assessment of capability was a striking predictor for the fundamental decision as to whether to provide dementia diagnostics in general practice (14% of total variance, R^2^). Availability of staff trained in dementia (26% of the total variance, R^2^) and especially personal background in geriatrics (12.2% of the total variance, R^2^) played a major role in perceptions of the ease or difficulty of diagnosing dementia in everyday practice. Awareness of regional support services proved to be an important factor in willingness to participate in treatment management in dementia patients (20.4% of the total variance, R^2^).

**Table 3:** Perceptions and willingness in general practice with reference to dementia diagnostics and treatment. Univariate linear regression, influential factors identified (*N* = 2266)

Independent variable (possible influential factor or predictor)	R^2^	R^2^ corrected	F (df = 1; 2264)	Coefficient of regression β	Significance	95% confidence interval	Standard error
*Dependent variable: Implementation of dementia diagnosis* *(“Do you provide dementia diagnostics in your practice?”)*							
Subjective capability in identifying dementia (Question 19)	0.14	0.14	369.04	0.322	0.000	0.289; 0.355	0.017
Challenge: Differential diagnosis (Question 10: Differential diagnoses between dementia and other forms of cognitive distinction, such as depression)	0.059	0.059	141.98	−0.21	0.000	−0.245; −0.176	0.018
Challenge: Treatment management (Question 10: successful treatment monitoring in dementia)	0.024	0.023	54.73	−0.119	0.000	−0.151; −0.088	0.016
Dementia patient treatment should always be left to specialists (Question 11)	0.09	0.09	224.55	−0.205	0.000	−0.232; −0.179	0.014
*Dependent variable: Subjective challenges to dementia detection as experienced in routine practice* *(“How would you generally rate the following statement: How easy or difficult is it for general practitioners to identify incipient dementia in patients during routine practice?”)*							
Practice staff trained in dementia (Question 18)	0.122	0.122	315.32	−0.609	0.000	−0.676; −0.542	0.034
Geriatrics qualification or adequate training (sociodemographics)	0.26	0.259	789.37	−0.304	0.000	−0.325; −0.283	0.011
*Dependent variable: Implementing treatment* *(“Are you usually involved in treating patients with dementia, or do you leave it all to neurologists or psychiatrists?”)*							
Assessment of cooperation between general practitioners and specialists in identifying and treating dementia patients (Question 12)	0.061	0.061	147.24	0.283	0.000	0.238; 0.329	0.023
Awareness of regional support structures in caring for dementia patients and their family members (Question 15)	0.204	0.204	580.84	0.557	0.000	0.512; 0.602	0.023
There are hardly any benefits to early dementia diagnosis due to the lack of treatment options (Question 11)	0.035	0.035	82.23	−0.17	0.000	−0.207; −0.134	0.019
Reluctance to diagnose dementia, therefore restrictions in treatment (Question 11)	0.023	0.022	52.97	−0.133	0.000	−0.169; −0.097	0.018

All the factors have been listed that show at least low explained variation according to Cohen [26]. Classification: low or weak explained variation |R^2^| = 0.02; medium or moderate explained variation |R^2^| = 0.13; high or strong explained variation |R^2^| = 0.26

## Discussion

### Main findings and comparison with prior work

The survey on 2266 general practitioners in Hesse and Baden-Württemberg confirmed the results from preliminary studies that around every fifth general practice did not provide dementia diagnostics [16, 17, 25]. A similar proportion of general practitioners left treatment management of dementia patients to specialists. In contrast, 51% did involve themselves in treating their dementia patients. The results also confirm other studies showing a number of challenges in routine general practice influencing the effectiveness of dementia diagnosis and treatment [7, 18–21, 27]. Low et al. concluded that the decision by doctors to use dementia diagnostics depended on, amongst other things, attitudes and opinions towards the disease and available treatment options, personal confidence in personal diagnostic and communication skills, psychosocial resources available to patients and family members, and medical knowledge and availability of support structures [21]. The study results reflect these points in more specific form.

Returning to the main research interest, the study was able to give a more accurate account of factors influencing the willingness of general practitioners to diagnose dementia on a consistent basis and involve themselves in treatment management:

- *Self-awareness in ability:* Respondents without dementia diagnostics in their own general practice more frequently expressed doubts as to their own subjective capabilities and saw more difficulty in identifying dementia in routine practice. Consequently, some general practitioners associated dementia diagnosis with a lack of treatment options (see Table 1; [4, 21, 28]). This led to a tendency for general practitioners to question the benefit of diagnosing dementia [7].
- *Integrating practice staff: *Less than half of general practices have staff trained in dementia, so practice staff members only played a minor role in early identification. Practice staff members trained in dementia were shown to be an influential factor in early identification of dementia. A qualitative interview study on the topic has shown that general practice staff would welcome closer involvement in identifying and treating dementia, and saw themselves as capable of assisting general practitioners especially in effectively identifying potential dementia patients [29, 30].
- *Training in geriatrics: *One striking finding was that fewer general practitioners without geriatric training performed screening examinations, had trained practice staff, or took notice of their staff members in identifying dementia compared to their trained counterparts. Regression analysis confirmed that training in geriatrics plays a major role in rapidly identifying dementia. Importantly, background knowledge in geriatrics may help successfully implement stabilization strategies in consultations with patients and their family members.
- *Cooperation with regional assistance and support services:* Every second respondent cooperated with regional supply services. By the same token, around half the general practitioners felt that they did not have enough awareness of the support services available. Awareness of regional support structures in caring for dementia patients and their family members were a relevant predictor for willingness to be involved in treatment management for dementia patients. Working with regional support services in dementia has mainly been focused on nursing services with far less attention given to services providing psychosocial stabilization for family caregivers. Systematic reviews in the international arena such as Mansfield et al. as well as Low et al. have confirmed that the lack of available or actively involved assistance and support networks in dementia care could be a limiting factor in medical care [19, 21].
- *Differential diagnosis and treatment paths:* Many of the respondents stated that differential diagnostic evaluation posed a challenge due to the time and cost constraints. Uncertainty in distinguishing dementia from other forms of cognitive impairment [31] was reinforced by lack of clarity in the diagnostic and treatment course as well as issues in working with specialists.

### Strengths and limitations

This survey drew on a large, heterogeneous sample population reaching into the depth and breadth of the primary care community; even so, a variety of limitations need to be considered in this survey. Two of these limitations were the regional recruiting focus in two federal states and the limited response rate. Apart from that, the danger of selection bias arises from general practitioners with a strong personal interest in the topic potentially disproportionately participating in this survey. The large number of doctors with geriatric training would suggest this to be the case. The response rate may have an effect on the relation between practitioners who are involved in the diagnosis of initial dementia and in the management of those patients because it is likely to assume that practitioners who have a negative opinion towards the therapeutic relevance of diagnosis may have also been not willing to respond to the questionnaire.

Nevertheless, the main findings—namely the associations between attitudes, knowledge, and confidence with regard to dementia diagnostics—should be interesting for the further improvement of primary care such as the development of disease management programs. Overall, further research should focus more on the targeted optimization of primary dementia care. It should try out application-oriented approaches for better dementia detection and care.

## Conclusion

The results tally with the general picture from other studies, according to which general practitioners are reluctant to diagnose dementia due to uncertainty on the topic and its diagnosis, low expectations of efficacy, and feared or perceived risks and stress [7, 18]. The use of resources available in the general practice also plays a major role [30]. This leads to negative consequences for the effectiveness of early identification and timely patient care.

The findings suggest that reinforcing additional training in geriatrics among general practitioners would be in order as the corresponding training contributes to sensitization towards dementia diagnostics. As a corollary, the value of early detection identification of dementia not only serves towards treatment but also more effective support in terms of quality of life [32]. Exploration into the possibilities of making geriatrics training more attractive for general practitioners would be warranted.

Apart from that, it would seem essential to involve general practitioners more intensively in cooperation and support structures focused on dementia [23, 33]. This refers not only to thorough knowledge of the support services available, but also to more intense cooperation with regional services. Referring patients and their family members to regional advice and support networks on time may lead to an improvement in patient care [6, 25] while also minimizing the risk of burnout amongst family caregivers [34]. Involving general practitioners more closely in these assistance structures [19, 33, 35] will also develop awareness that the importance of effective and early diagnosis cannot be overestimated. Scientifically supported model projects are already striving to strengthen the integration of general practice-based dementia care in regional advice and support networks [6, 36, 37].

It would also be of benefit to involve general practice staff more closely and thoroughly in the identification and treatment of dementia patients [29, 30]. Employees with corresponding training would be able to assist general practitioners effectively in identifying the symptoms of dementia early on and in stabilizing patients and their family members [6].

## Supplementary Information

Questionnaire (German)

## Footnotes

1 Further training in geriatrics encompasses prevention, diagnosis, and treatment of geriatric diseases. Focal points in this training include cognitive neuropsychological disorders such as dementia and depression as well as symptomatology and functional importance in age-related changes and specific diseases. This entails acquiring knowledge on performing geriatric assessments including tests for cognitive ability, behavior, and emotional condition. Consultation with regard to sociomedical as well as care and support-related issues also plays a role.

2 Response rate by state: 25% (950) for Hesse, 20% (1316) for Baden-Württemberg.
