# Supplementary material for: Factors influencing general practitioners’ perception of and attitude towards dementia diagnostics and care—results of a survey among primary care physicians in Germany
Source: Wien Med Wochenschr. 2021 Jan 14;171(7):165–73. doi: 10.1007/s10354-020-00803-9 (PMC8057991; doi:10.1007/s10354-020-00803-9)
Supplement: Supplementary file 1 — Questionnaire (German) [file 10354_2020_803_MOESM1_ESM.pdf]

1. Was sind Ihrer Erfahrung nach häufige Anzeichen einer beginnenden Demenz? Bitte nennen Sie drei wichtige Symptome.

____

____

2. Wenn Sie an die Patienten denken, bei denen Sie in den letzten Jahren auf eine (beginnende) Demenz gestoßen sind oder einen (unspezifischen) Demenzverdacht hatten: Was waren die ersten Hinweise, durch die Sie auf das Vorliegen einer möglichen Demenz aufmerksam wurden? (Mehrfachangabe möglich)

◯ Beschwerden des Patienten selbst ◯ Hinweise Angehöriger ◯ Hinweise des Praxispersonals
◯ Eigene Beschäftigung mit dem Patienten

◯ Sonstige, und zwar: ____________________________________

3. Bieten Sie in Ihrer Praxis eine Demenzdiagnostik an?

◯ Ja ◯ Nein, inzwischen nicht mehr (abgeschafft) ◯ Nein, noch nie

- Falls ‚Nein': Bitte direkt zu Frage 6 übergehen -

4. Welche Testverfahren führen Sie in der Praxis zur Früherkennung von Demenz durch? (Mehrfachangabe möglich)

◯ Mini-Mental-Status-Test (MMST) ◯ DemTect ◯ Uhrentest ◯ Montreal Cognitive Assessment (MoCA)
◯ Geldzähltest nach Nikolaus ◯ Test zur Früherkennung von Demenzen mit Depressionsabgrenzung (TFDD)

◯ Sonstige, und zwar: ____________________________________

5. Kommen Demenztests bei Ihnen ausschließlich im Verdachtsfall bzw. zur Verlaufskontrolle zum Einsatz oder auch zu Screeningzwecken?

◯ Ausschließlich im Verdachtsfall bzw. zur Verlaufskontrolle ◯ Auch zu Screeningzwecken
◯ Überwiegend im Rahmen des Geriatrischen Basisassessments (GBA)

6. Überweisen Sie Patienten mit Verdacht auf Demenz in der Regel zur weiteren Abklärung? (Mehrfachangabe möglich)

◯ Ja, zum niedergelassenen Neurologen/Psychiater
◯ Ja, zur Spezialambulanz für Gedächtnisstörungen (vorausgesetzt, eine solche befindet sich in einer für Patienten zumutbaren Entfernung)
◯ Überweise in der Regel nicht

7. Sind Sie in der Regel in die Therapie von Demenzpatienten eingebunden, oder überlassen Sie dies ausschließlich dem neurologischen bzw. psychiatrischen Spezialisten?

◯ In der Regel in Therapie eingebunden ◯ Nur in Einzel-/Ausnahmefällen ◯ Überlasse ausschließlich Spezialist

Hinweis: ***Die nachfolgenden Fragen beziehen sich nicht unmittelbar auf Ihre Praxis, sondern auf Ihre generellen Einschätzungen hinsichtlich der hausärztlichen Demenzversorgung.***

8. Wie würden Sie das generell einschätzen: Wie leicht oder schwer ist es im Alltag für den Hausarzt, bei Patienten eine beginnende Demenz zu erkennen?

◯ Sehr schwer ◯ Eher schwer ◯ Eher leicht ◯ Sehr leicht

9. Gelegentlich hört und liest man, dass in vielen Hausarztpraxen die Diagnose Demenz häufig erst in relativ fortgeschrittenem Stadium gestellt wird. Woran liegt das Ihrer Ansicht nach? Einige Stichpunkte genügen.

____

____

____

10. Die hausärztliche Demenzversorgung bringt verschiedene Herausforderungen mit sich. Bitte geben Sie jeweils an, für wie groß Sie die folgenden Herausforderungen tendenziell halten.

	Sehr groß	Eher groß	Eher gering	Sehr gering
Differenzialdiagnostische Abgrenzung einer Demenz von anderen Formen kognitiver Beeinträchtigungen (z.B. Depression)	◯	◯	◯	◯
Sicherstellung der Akzeptanz und Kooperation des Patienten bei der Durchführung von Demenztests	◯	◯	◯	◯
Aufklärung des Patienten über die Diagnose und Sicherstellung der Compliance	◯	◯	◯	◯
Kommunikation mit pflegenden Angehörigen	◯	◯	◯	◯
Erfolgreiche therapeutische Begleitung von Demenzerkrankten	◯	◯	◯	◯
Beratung und Versorgung von Demenzpatienten, die sich kostendeckend für die Praxis darstellt	◯	◯	◯	◯

11. Hier stehen verschiedene Aussagen von Allgemeinmedizinern, die im Rahmen einer Interviewstudie erfasst wurden. Wie stark stimmen Sie der jeweiligen Aussage zu?

	Stimme voll und ganz zu	Stimme eher zu	Stimme eher nicht zu	Stimme gar nicht zu
„Patienten mit Demenz sollten maßgeblich durch Fachärzte wie Neurologen oder Psychiater betreut werden, nicht durch den Hausarzt.“	○	○	○	○
„Im Bereich Demenz fehlt es an wirtschaftlichen Anreizen, warum ein Hausarzt sich damit befassen sollte – die Honorierung müsste deutlich besser sein.“	○	○	○	○
„Es bringt im Prinzip kaum Vorteile, eine Demenz im Frühstadium zu erkennen, da die therapeutische Konsequenz unzureichend ist.“	○	○	○	○
„Die Diagnose Demenz bedeutet für viele Patienten einen Angriff auf die Entscheidungsautonomie und erzeugt mir gegenüber starke Abwehrhaltungen, was die weitere Patientenversorgung einschränkt.“	○	○	○	○

12. Wie beurteilen Sie aus eigener Erfahrung die Zusammenarbeit zwischen Hausärzten und Fachärzten (Neurologen, Psychiater), wenn es um die Erkennung und Versorgung von Demenzpatienten geht?

○ Sehr gut ○ Eher gut ○ Eher schlecht ○ Sehr schlecht

13. Wie beurteilen Sie die Zusammenarbeit zwischen Hausärzten und Alten- bzw. Pflegeheimen, wenn es um die Erkennung und Versorgung von Demenzpatienten geht?

○ Sehr gut ○ Eher gut ○ Eher schlecht ○ Sehr schlecht

14. Bei der Zusammenarbeit zwischen Hausärzten und Fachärzten im ambulanten Bereich zur Erkennung und Versorgung von Demenzpatienten können verschiedene Schwierigkeiten auftreten. Wie häufig treten die folgenden Schwierigkeiten Ihrer Erfahrung nach auf?

Neurologische/psychiatrische Fachärzte sind für die Patienten schlecht erreichbar.

◯ Häufig ◯ Gelegentlich ◯ Selten ◯ Nie

Es fehlt im Praxisalltag die erforderliche Zeit, um sich mit den Kollegen über die meist komplexen Patientenprobleme auszutauschen.

◯ Häufig ◯ Gelegentlich ◯ Selten ◯ Nie

Die niedergelassenen neurologischen/psychiatrischen Fachärzte sind auf lange Zeit ausgebucht.

◯ Häufig ◯ Gelegentlich ◯ Selten ◯ Nie

Man muss lange warten, bis die neurologischen/psychiatrischen Fachärzte ihre Befunde weiterleiten.

◯ Häufig ◯ Gelegentlich ◯ Selten ◯ Nie

Neurologische/psychiatrische Fachärzte informieren den Hausarzt nicht ausreichend über die von ihnen durchgeführten Untersuchungen, Ergebnisse und/oder gestellte Diagnosen.

◯ Häufig ◯ Gelegentlich ◯ Selten ◯ Nie

Neurologische/psychiatrische Fachärzte greifen zu nicht nachvollziehbarer oder nicht für Demenz zugelassenener Medikation von Demenzpatienten.

◯ Häufig ◯ Gelegentlich ◯ Selten ◯ Nie

Die Patienten werden vom neurologischen/psychiatrischen Facharzt nicht ausreichend über ihre Situation aufgeklärt und kehren aus Unsicherheit zum Hausarzt zurück.

◯ Häufig ◯ Gelegentlich ◯ Selten ◯ Nie

Es gibt zu wenige neurologische/psychiatrische Praxen in der Nähe.

◯ Häufig ◯ Gelegentlich ◯ Selten ◯ Nie

15. Was würden Sie sagen: Wie gut kennen Sie sich mit den regionalen Versorgungsstrukturen zur Betreuung von Demenzpatienten aus? (z.B. Demenz-Netzwerke, Arbeit und Service der Pflegestützpunkte)

◯ Sehr gut ◯ Eher gut ◯ Eher nicht so gut ◯ Gar nicht

16. Arbeiten Sie bei der Betreuung von Demenzpatienten mit anderen Einrichtungen/Diensten zusammen?

◯ Ja, mit folgenden Einrichtungen/Diensten:

____

◯ Nein

17. Für wie hilfreich halten Sie die folgenden Maßnahmen, um die Früherkennung von Demenz in der Hausarztpraxis wirksam zu verbessern?

Konsequentes und regelmäßiges Screening bei älteren Patienten ab einer bestimmten Altersgrenze (z.B. ab 70 Jahren)

◯ Sehr hilfreich ◯ Eher hilfreich ◯ Weniger bzw. gar nicht hilfreich

Etablierung eines strukturierten Diagnose- und Therapiealgorithmus, der Hausärzten ein Verhaltens- und Orientierungsschema zur systematischen Demenzerkennung gibt

◯ Sehr hilfreich ◯ Eher hilfreich ◯ Weniger bzw. gar nicht hilfreich

Verstärkte Zusammenarbeit zwischen Hausärzten und regionalen Unterstützungsnetzwerken (z.B. Demenz-Netzwerke, Pflegestützpunkte)

◯ Sehr hilfreich ◯ Eher hilfreich ◯ Weniger bzw. gar nicht hilfreich

Bessere Honorierung der Hausärzte bei demenzbezogenen Beratungs-, Diagnose- und Versorgungsleistungen

◯ Sehr hilfreich ◯ Eher hilfreich ◯ Weniger bzw. gar nicht hilfreich

Verstärkte Schulung der Praxismitarbeiter hinsichtlich der Früherkennung von Demenz sowie Demenzdiagnostik

◯ Sehr hilfreich ◯ Eher hilfreich ◯ Weniger bzw. gar nicht hilfreich

18. Haben Sie in Ihrer Praxis Mitarbeiter, die auf dem Gebiet Demenz speziell geschult sind? (Gemeint ist der Besuch entsprechender Fortbildungen.)

◯ Ja ◯ Nein

19. Wie kompetent fühlen Sie sich, wenn es um das Thema Früherkennung von Demenz im Allgemeinen geht?

◯ Sehr kompetent ◯ Eher kompetent ◯ Eher nicht so kompetent

20. Wie groß wäre Ihr Interesse, an einer Fortbildung zum Thema Demenz teilzunehmen?

◯ Sehr groß ◯ Eher groß ◯ Eher nicht so groß ◯ Kein Interesse

21. Unabhängig davon, ob die Teilnahme an einer Fortbildung zum Thema Demenz für Sie in Frage kommt oder nicht: Welche der folgenden Themengebiete würden Sie im Rahmen einer solchen Veranstaltung interessieren? Bitte stufen Sie ab.

Kein Interesse — Sehr großes Interesse

	0	1	2	3	4
Demenz-Leitlinien	◯	◯	◯	◯	◯
Schritte hin zum Demenzverdacht und weiteres Vorgehen	◯	◯	◯	◯	◯
Diagnostik und Testverfahren (Durchführung des Diagnostikprozesses und Interpretation)	◯	◯	◯	◯	◯
Therapieformen (insbesondere Antidementiva und andere Psychopharmaka)	◯	◯	◯	◯	◯
Gesprächsführung mit an Demenz Erkrankten (z.B. Validationstechniken)	◯	◯	◯	◯	◯
Kommunikation mit pflegenden Angehörigen und deren Einbindung (Empfehlungen, Vermeidung einer Überlastung von Angehörigen etc.)	◯	◯	◯	◯	◯
Aufklärung über Diagnose und Sicherstellung der Compliance	◯	◯	◯	◯	◯
Regionale Versorgungs- und Betreuungsangebote bei Demenz (Pflegestützpunkte, Demenz-Netzwerke)	◯	◯	◯	◯	◯
Rechtliche Aspekte (Vorsorgevollmacht, Betreuung, „Autofahren“)	◯	◯	◯	◯	◯

22. In welcher Form würden Sie eine solche Fortbildung bevorzugt in Anspruch nehmen?

◯ Als Fortbildung vor Ort ◯ Als Online-Fortbildung ◯ Als Fortbildungs-DVD ◯ Als Fortbildungs-Buch oder -Heft

Sonstiges : ______________________________

Nun bräuchten wir noch einige statistische Angaben von Ihnen. Wie auch der Rest des Fragebogens werden diese Informationen natürlich streng vertraulich behandelt und anonymisiert.

Geschlecht:

◯ Männlich ◯ Weiblich ◯ Divers

Ihr **Alter**: _____

Ihr **Bundesland**:

◯ Hessen ◯ Baden-Württemberg

Wo befindet sich Ihre Praxis? In einer Gemeinde/Stadt mit . . .

◯ mehr als 100.000 ◯ 20.000-100.000 ◯ 5.000 bis 20.000 ◯ unter 5.000 Einwohnern

Welches Niederlassungsmodell beschreibt Ihre Praxis am besten?

◯ Einzelpraxis (Sie sind der/die einzige Arzt/Ärztin) ◯ Einzelpraxis mit angestellten Ärzten
◯ Gemeinschaftspraxis ◯ Medizinisches Versorgungszentrum

Wie viele Ärzte sind in Ihrer Praxis tätig?

◯ 1 Arzt ◯ 2 Ärzte ◯ 3 Ärzte ◯ Mehr als drei Ärzte

Wie viele Patienten behandelt Ihre Praxis im Quartal?

◯ 500-750 ◯ 751-1000 ◯ 1001-1500 ◯ Mehr als 1500

Verfügen Sie über die **Fachkunde Geriatrie** oder eine adäquate Weiterbildung?

◯ Ja ◯ Nein

<u>Vielen Dank für Ihre Teilnahme!</u>

Sollten Sie diese Befragung schriftlich ausgefüllt haben, nutzen Sie bitte den von uns beigefügten Rückumschlag.

Was möchten Sie uns noch mitteilen?
Hier finden Sie Platz für Anregungen, Kommentare und Kritik.

____

____

____
